# Examining Cough’s Role and Relief Strategies in Interstitial Lung Disease

**DOI:** 10.3390/jcm14010291

**Published:** 2025-01-06

**Authors:** Chee Yao Lim, Sanam Wasim Khan, Tarek Alsibai, Gayathri Sathiyamoorthy

**Affiliations:** 1 Corewell Health, Grand Rapids, MI 49503, USA; tarek.alsibai@corewellhealth.org (T.A.); gayathri.sathiyamoorthy@corewellhealth.org (G.S.); 2 College of Human Medicine, Michigan State University, East Lansing, MI 48824, USA; 3 CMH Lahore Medical College, Lahore 54810, Pakistan; sanamhumayun13@gmail.com

**Keywords:** cough, interstitial lung disease, idiopathic pulmonary fibrosis, treatment, update

## Abstract

Chronic cough is a distressing and prevalent symptom in interstitial lung disease (ILD), significantly impairing quality of life (QoL) and contributing to disease progression, particularly in idiopathic pulmonary fibrosis (IPF). It is associated with physical discomfort, psychological distress, and social isolation and is often refractory to conventional therapies. The pathophysiology of cough in ILD is complex and multifactorial, involving neural hypersensitivity, structural lung changes, inflammatory processes, and comorbid conditions such as gastroesophageal reflux disease (GERD). Evaluating cough in ILD relies on subjective and objective tools to measure its severity, frequency, and impact on daily life, although standardization of these measures remains challenging. Management strategies span pharmacological interventions, including neuromodulators such as opiates, antifibrotic agents, pharmacologic and surgical GERD treatments, and non-pharmacological approaches like behavioral therapies, cough suppression techniques, and pulmonary rehabilitation and physiotherapy. Emerging treatments, such as P2X3 receptor antagonists and airway hydration therapies, offer promising avenues but require further investigation through robust clinical trials. This review aims to demonstrate the importance of addressing cough in ILD as a significant symptom and present objective and subjective methods of quantifying coughs, while providing insights into effective and emerging therapeutic options. By highlighting these potential therapies, we hope to guide healthcare practitioners in considering them through a thorough evaluation of benefits and risks on a case-by-case basis, with relevance both in the U.S. and internationally.

## 1. Cough in ILD

Interstitial lung disease (ILD) encompasses a heterogeneous group of pulmonary disorders that are marked by inflammation and fibrosis of the lung interstitium, leading to progressive respiratory dysfunction, which includes chronic cough. Chronic cough, a prevalent and debilitating symptom in ILD, significantly impacts quality of life [1,2,3] and contributes to physical discomfort, psychological distress, and social isolation, all of which pose substantial clinical management challenges [4]. Chronic cough in ILD is a significant outcome measure of the management of ILD [3], which is often refractory to conventional therapeutic interventions. The cough severity was found to be higher in patients with idiopathic pulmonary fibrosis (IPF) compared to non-IPF fibrotic ILD, and the baseline cough severity was independently associated with disease progression and reduced transplant-free survival [5,6].

The pathophysiology of cough in ILD is multifactorial and only partially understood. It involves complex interactions between mechanical alterations in the lung architecture, the activation of neural pathways, and inflammatory processes. Factors such as fibrotic remodeling, increased airway sensitivity, and coexistent conditions like gastroesophageal reflux disease (GERD) and upper airway cough syndrome further complicate the clinical picture. 

Current treatment approaches for cough in ILD are often empirical and yield variable results, highlighting a significant unmet clinical need [7]. Therapeutic options range from pharmacological interventions, including antitussives, neuromodulators, and anti-inflammatory agents, to non-pharmacological measures, such as pulmonary rehabilitation and cough suppression techniques. Recent advances in developing antifibrotic therapies and targeted neuromodulators offer promising avenues for improved symptom control. The efficacy and safety profiles of these treatments necessitate further exploration through robust clinical trials.

**Figure 1:**
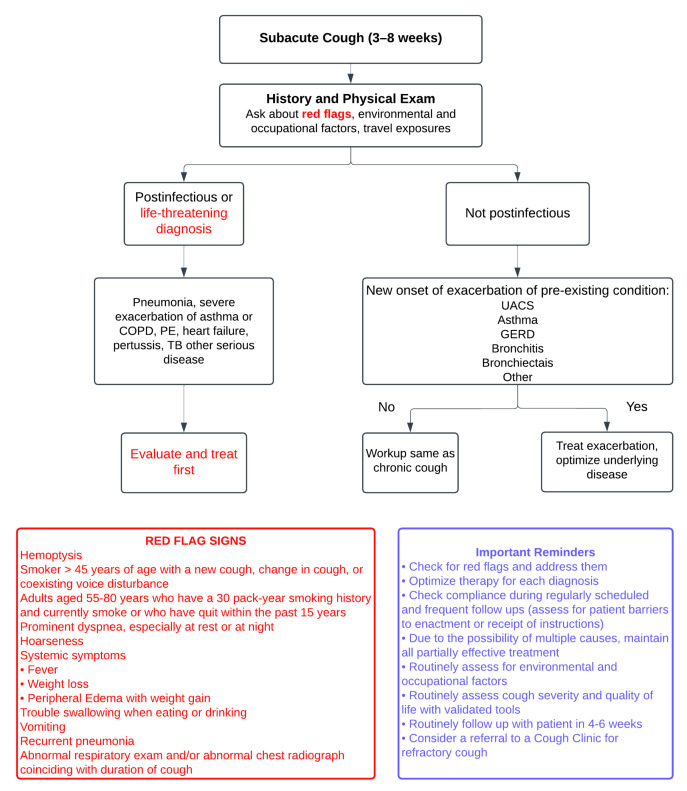
The figure illustrates a systematic approach to diagnosing subacute cough (3–8 weeks), beginning with a comprehensive history and physical examination to identify “red flags” such as hemoptysis, significant weight loss, recurrent pneumonia, or abnormal imaging findings, while also considering environmental, occupational, and travel exposures. Postinfectious or life-threatening conditions, including pneumonia, severe asthma or COPD exacerbations, pulmonary embolism, or tuberculosis, require immediate evaluation and treatment. For non-postinfectious causes, such as exacerbations of chronic conditions like upper airway cough syndrome (UACS), asthma, or GERD, management focuses on optimizing the underlying disease. In cases without exacerbation, the workup follows that of chronic cough. Red flag signs are clues to potential life-threatening conditions. Adapted from: Irwin RS et al., 2017 [8].

**Figure 2:**
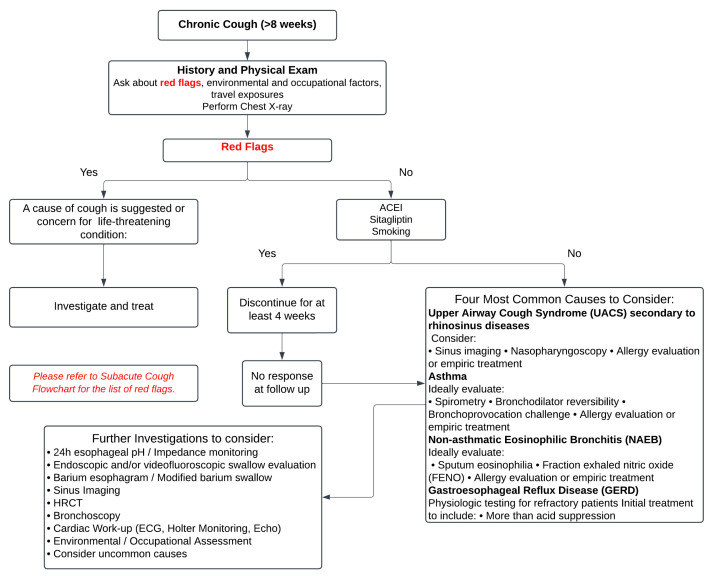
The figure presents a structured approach to the evaluation and management of chronic cough (≥8 weeks), beginning with a detailed history, physical examination, and chest X-ray to identify potential “red flags” suggestive of life-threatening or serious conditions, which warrant immediate investigation and treatment. In the absence of red flags, common causes such as upper airway cough syndrome (UACS), asthma, gastroesophageal reflux disease (GERD), or non-asthmatic eosinophilic bronchitis (NAEB) should be systematically considered and managed. For unresolved cases after 4 weeks of treatment, further investigations such as 24-h pH monitoring, spirometry, allergy testing, and HRCT may be necessary. It emphasizes a stepwise evaluation to address underlying causes while minimizing unnecessary interventions. Adapted from: Irwin RS et al., 2017 [8].

This review aims to elucidate the current understanding of cough’s epidemiology, pathophysiology, and impact on ILD, while emphasizing the need for standardizing the approach to diagnosing coughing that is specific to ILD/IPF, distinguishing it from chronic coughs of other etiologies. Furthermore, it highlights advancements in measuring and monitoring cough in ILD patients and explores updated and solidified treatment options. By synthesizing the contemporary evidence, this review provides a comprehensive resource to guide clinicians and researchers in improving the management of cough and enhancing patient outcomes in ILD. Figure 1 and Figure 2 illustrate a systematic approach to the diagnosis and investigation of patients presenting with subacute and chronic cough [8].

## 2. Measuring Cough in ILD

The assessment of cough, particularly in terms of its severity, frequency, and impact on health-related quality of life (HRQOL), is essential in clinical practice and research. Among the key tools that are used to evaluate cough in ILD patients are the Leicester Cough Questionnaire (LCQ), the Cough Quality of Life Questionnaire (CQLQ), and the Cough Visual Analog Scale (VAS).

The LCQ is a widely accepted measure of cough severity and its impact on quality of life [9]. It is a 19-item, self-administered questionnaire that assesses cough in three physical, psychological, and social domains [Appendix A]. The LCQ has been particularly valuable in patients with ILD, because it captures the multidimensional impact of chronic cough, which often worsens over time in progressive diseases like IPF [10]. By providing a reliable measure of cough severity and its effect on everyday activities, the LCQ is used to track disease progression and evaluate the effectiveness of interventions [11]. Improvements in LCQ scores have been associated with a reduction in cough severity, reflecting an overall improvement in HRQOL. Similarly, a lower LCQ score is independently linked to an increased risk of respiratory hospitalization, death, and the need for lung transplantation [11].

Similarly, the CQLQ focuses on how chronic cough affects a patient’s daily life. This tool delves into different aspects of well-being, including physical discomfort, emotional health, and the social implications of persistent cough [Appendix B] [12]. In ILD, where cough is often refractory to treatment, the CQLQ is a valuable outcome measure to determine how well therapeutic interventions address cough-related symptoms and their psychosocial burden. Clinicians and researchers use this tool to gauge the symptom burden and understand the treatment response more holistically by assessing changes in HRQOL.

The VASc is another tool that is used to assess cough in ILD, especially for its simplicity and ease of use. It is a unidimensional scale, where patients rate their perceived cough severity on a continuum, usually a 100 mm line ranging from “no cough” to “worst cough imaginable” [Appendix C]. The VASc is quick to administer and can be highly useful in clinical settings for both initial assessments and follow-up visits. The cough VAS can be applied in ILD patients alongside more detailed, objective tools like the LCQ and CQLQ to provide a rapid snapshot of symptom severity [13]. It is often employed to monitor treatment responses over time, as shifts in VASc scores can indicate changes in cough severity that correlate with HRQOL improvements [14]. A recent study looked into the VASc in predicting survival and identified significant quality of life differences in patients with IPF, with a clinically meaningful cutoff score of 30 mm [15]. More studies would be helpful to validate the VASc among ILD patients [16].

The St. George’s Respiratory Questionnaire (SGRQ) assesses HRQOL in patients with chronic respiratory diseases, including ILD [Appendix D] [17,18]. While it has been validated and utilized in various studies involving ILD patients, the SGRQ only partially addresses cough. Cough is included as part of its broader assessment of respiratory symptoms rather than being a central focus. As a result, the SGRQ provides insight into how cough may contribute to the overall symptom burden. Still, it does not explicitly measure cough severity or its detailed impact on quality of life. 

The Living with Pulmonary Fibrosis (L-PF) questionnaire is a relatively new patient-reported outcome (PRO) measure, developed to assess symptoms and quality of life in patients with progressive fibrosing ILD (PF-ILDs) [Appendix E]. The L-PF is an adaptation of the original Living with Pulmonary Fibrosis (L-IPF) questionnaire and includes two modules: Symptoms and Impacts. It focuses on crucial symptom domains, such as dyspnea, cough, and fatigue, and uses a 24 h recall period to capture daily fluctuations in these symptoms. Validation studies have confirmed the relevance of the L-PF for patients with various forms of PF-ILDs, demonstrating good face validity and responsiveness to change. This makes it a valuable tool for both clinical research and potentially for routine clinical practice, providing insights into the symptom burden and quality of life impacts for patients [19,20].

These tools—the LCQ, CQLQ, SGRQ, and cough VASc—offer complementary approaches to measuring cough and its impact on patients with ILD. While the LCQ and CQLQ provide a more comprehensive assessment of cough’s multidimensional effects, the VAS offers a straightforward and immediate measure of the symptom severity, allowing for a detailed understanding of the patient’s experience and tracking of the treatment efficacy. 

The measures discussed are subjective patient-reported outcomes, which primarily focus on how cough symptoms affect quality of life [21]. Attempts at validating the tools have been made and correlate well with the objective cough severity [13]. A limitation of these tools is that they do not directly measure cough severity but address it indirectly through their impact on daily functioning. This distinction is important, because improvements in a patient’s perception of their cough may result from actual cough suppression but also from the central neuromodulatory effects of treatments. These treatments could include opioids, pregabalin, and gabapentin, which reduce the unpleasantness of the cough experience [7]. 

Several modern objective cough measuring tools such as the Leicester Cough Monitor (LCM) and VitaloJAK^TM^ are commercially available. These are primarily used in clinical trials to validate subjective cough measurements across various disease-specific cough conditions [22]. Although direct comparisons between these tests are lacking, their results are relatively consistent [22].

## 3. GERD and Cough in ILD

Over the past 10–15 years, evidence has highlighted the complex relationship between GERD and cough in ILD [23,24]. GERD is commonly observed in patients with ILD, particularly in those with idiopathic pulmonary fibrosis (IPF), where the prevalence of reflux can be as high as 90% [24,25]. The connection between GERD and cough in ILD is thought to stem from silent microaspiration of gastric contents into the lower airways. This causes or exacerbates lung inflammation, which may worsen the underlying disease and contribute to a chronic, persistent cough [24].

Several studies have suggested that GERD may act as both a trigger and perpetrator of cough in ILD patients. Aggressive GERD management, including lifestyle changes or using proton pump inhibitors (PPIs) or Histamine 2 receptor blockers, may help control cough and potentially slow the disease progression [26,27]. However, the benefit of antireflux therapies in improving overall ILD outcomes remains controversial, with some studies showing improvement in cough symptoms but limited evidence of a clear impact on long-term disease progression or survival [28].

One key study by Raghu et al. (2006) [29] explored the high prevalence of GERD in patients with IPF and suggested that antireflux treatment could reduce chronic cough, but randomized controlled trials have not consistently confirmed a clear therapeutic benefit. Additionally, non-acid reflux and silent aspiration—conditions where patients experience reflux without typical GERD symptoms—complicate the diagnosis and management of cough in ILD [30]. These forms of reflux may still contribute to cough and disease progression but are more challenging to detect and treat. Thus, conventional GERD therapies alone may not be sufficient for all ILD patients with cough [31].

Moreover, in recent years, the role of surgical interventions, such as fundoplication, has been investigated as a more definitive approach to controlling GERD in ILD patients [32]. Although the causal relationship is not well established, risk factors include cough, and an association with aspiration and the worsening of IPF is a suggested theory [32,33]. There are limited data stating that repairing hiatal hernia would improve outcomes among patients in IPF or symptomatic controls [34]. We believe that with cough as a predictor of mortality among patients with IPF, treating cough effectively by means of acid suppression and surgical correction of anatomical anomalies such as hiatal hernia would be a conjugate of mortality benefit. More studies would need to be carried out. While some studies suggest that surgical intervention may help reduce the incidence of reflux-related cough and microaspiration [35], the risks associated with surgery, particularly in patients with advanced lung disease, remain a concern. More research is needed to determine which subsets of ILD patients are most likely to benefit from GERD management strategies, both pharmacological and surgical.

## 4. Opiates as Antitussive Treatment in ILD-Associated Cough

Opiates have long been recognized for their potent antitussive (cough-suppressing) properties. They work by directly suppressing the cough reflex through mu receptors in the brainstem. Their use in treating chronic refractory cough, including cough associated with ILD, has received significant attention in recent years.

In ILD, where chronic cough can be debilitating, low-dose opioids have been explored as a therapeutic option to improve quality of life. Morphine decreased the cough frequency, improved patients’ comfort, and reduced the distress associated with persistent coughing. Other studies have supported these findings, indicating that low-dose opioids (5 mg controlled-release morphine sulfate (MST)) can be a valuable adjunct for symptom control in refractory cough when other options are ineffective [36]. In a randomized, double-blind, placebo-controlled crossover trial, a nalbuphine extended-release tablet (NAL ER) was shown to significantly reduce cough in IPF [37]. This drug is currently not available in the United States. Table 1 provides an overview of studies evaluating the role and efficacy of opioid therapy in managing cough among patients with IPF.

Despite the potential benefits, the use of opiates in ILD patients is not without challenges. Although low-dose morphine has a good safety profile, it does carry a risk of side effects, including nausea, vomiting, constipation, and, importantly, respiratory depression, which can be particularly concerning in patients with already impaired lung function [36,38]. This risk requires careful consideration and monitoring when prescribing opioids to ILD patients, particularly those with more advanced disease and age. As a result, opiates are often reserved for patients with severe cough who have not responded to other therapeutic options.

Codeine, a weaker opioid, is sometimes used to manage chronic cough but tends to be less effective than morphine in controlling severe cases. While it is more commonly prescribed due to its milder side effect profile, its efficacy in ILD-related cough is less well established [39]. The balance between symptom relief and minimizing side effects remains a crucial consideration in the use of any opioid for cough management in ILD.

## 5. Role of Neuromodulators in Cough Associated with ILD

In recent years, neuromodulators such as gabapentin and pregabalin have emerged as potential therapeutic options for treating refractory cough. Since cough in ILD can be refractory, there has been significant research interest in exploring novel approaches targeting the neural pathways that are involved in cough reflex hypersensitivity. Gabapentin and pregabalin, traditionally used to treat neuropathic pain, are believed to modulate the excitability of sensory nerves that trigger cough, offering relief in cases where other treatments have failed.

Gabapentin, in particular, has been studied more extensively for its role in reducing chronic cough. Several randomized controlled trials, including studies conducted in non-ILD populations, have shown that gabapentin can significantly minimize the cough frequency and severity by suppressing the heightened sensitivity of the cough reflex. These findings have led to its off-label use in patients with ILD who experience debilitating coughs. A notable study by Ryan et al. (2012) demonstrated that gabapentin reduced cough symptoms in patients with chronic cough of various etiologies, including those related to lung disease [40]. However, the specific evidence relating to ILD remains limited, and more research is needed to fully understand its efficacy in this population.

Pregabalin, a related neuromodulator, has also been explored as a treatment for chronic cough [41]. Like gabapentin, pregabalin works by reducing neuronal excitability and has shown promise in reducing the cough frequency in small trials. While the available data on pregabalin in ILD-related cough are sparse, its similar mechanism of action suggests that it could be a viable option for patients who do not respond to other treatments [42]. Both drugs are typically well tolerated, although side effects such as drowsiness, dizziness, and fatigue may limit their use in some patients. Starting at a lower dose and titrating up can overcome these side effects and improve tolerance.

Duloxetine is a serotonin norepinephrine reuptake inhibitor that is used for various psychiatric conditions and chronic pain. There is currently an active trial evaluating the role of duloxetine in refractory chronic cough by inhibiting the activity of the transient receptor potential vanilloid 1 (TRPV1) channel [43]. The study aims to investigate duloxetine’s efficacy in reducing cough by modulating central hypersensitivity pathways, potentially offering a novel therapeutic option for this challenging condition.

## 6. Macrolides

Macrolides, particularly azithromycin, have been studied for their potential to treat chronic cough in various respiratory conditions, including IPF and chronic productive cough. In IPF, a randomized controlled trial found no significant improvement in cough-related quality of life or objective cough frequency after 12 weeks of azithromycin therapy compared to a placebo, despite its known anti-inflammatory effects [44]. However, in patients with chronic productive cough of unknown origin, azithromycin demonstrated a marked improvement in cough-related quality of life (LCQ scores), sputum volume, and sputum color after a 12-week course of treatment, especially in those with neutrophilic airway inflammation [45]. These findings suggest that macrolides may benefit specific chronic cough subgroups, but their efficacy in IPF-related cough remains uncertain.

## 7. Inhaled Corticosteroids 

Oral and inhaled corticosteroids are widely used to treat a variety of lung conditions and their symptoms. A small non-randomized study looked into the positive effect of corticosteroids in controlling cough in IPF patients and showed a significant reduction in VAS score [46]. However, there are no studies to date that improve the quality of life or survival among patients with cough in IPF [47]. The small benefit of improved symptoms needs to be weighed against the larger side effect profile of chronic steroid use. Among patients with Sjogren Syndrome-related ILD, such as nonspecific interstitial pneumonia (NSIP) and lymphocytic interstitial pneumonia (LIP), corticosteroids are not beneficial in improving the course of disease, nor in reducing cough [48].

Inhaled corticosteroids (ICSs), like budesonide and fluticasone, have been used as alternatives or adjuncts to oral steroids, particularly in patients with milder disease or airway hyperreactivity. Although some studies suggest that ICSs can reduce cough and improve quality of life in subset of ILD patients (sarcoidosis), their efficacy in improving lung function or preventing long-term disease progression is limited [49,50,51]. Inhaled corticosteroids (ICSs) have been evaluated for their effectiveness in treating chronic cough associated with COVID-19. A study published in March 2024 investigated the use of ICSs in patients experiencing persistent cough following COVID-19 infection during the Omicron variant outbreak. The findings indicated that while ICS treatment did not significantly alleviate cough symptoms, it did improve impaired the lung function in these individuals [52]. 

## 8. Pirfenidone in IPF and Other ILDs 

Pirfenidone is an antifibrotic and anti-inflammatory agent that inhibits the TGF-B and TNF-a pathways; it is used for the treatment of IPD to slow disease progression [53]. It has demonstrated potential in managing cough associated with IPF and fibrosis related to COVID-19. In an international multicenter study, pirfenidone reduced the objective 24 h cough frequency by 34% after 12 weeks, showing both objective improvements in cough counts and subjective enhancements in cough-related QoL measures [54,55]. Another study further supported these findings, reporting improvement in patient-reported symptoms, including cough and general well-being, after 12 months of treatment with pirfenidone, making it a promising option for cough management in fibrotic lung diseases [56,57].

Both the RELIEF trial and Maher et al.’s 2019 study explored the efficacy of pirfenidone in treating patients with progressive fibrosing interstitial lung diseases (PF-ILD). In both trials, there were no significant differences in quality of life improvements, as measured by the SGRQ. Despite promising to slow the decline of the forced vital capacity (FVC), the studies did not demonstrate substantial improvements in symptom relief, particularly regarding cough or overall respiratory symptoms, as captured by the SGRQ [58,59].

## 9. Nintedanib in IPF and PPF

Nintedanib is a tyrosine kinase inhibitor targeting the vascular endothelial growth factor, platelet-derived growth factor, and fibroblast growth factor pathways and is approved for treating IPF and slowing the disease progression [60]. In studies evaluating the role of nintedanib in IPF, connective-tissue-disease-associated interstitial lung disease (CTD-ILD), and progressive pulmonary fibrosis (PPF), cough-related parameters such as those measured by the SGRQ, L-PF, and LCQ were primary and secondary outcomes, respectively. These studies focused on slowing lung function decline, but improvements in respiratory-related quality of life, including cough, were also observed. While nintedanib’s main impact was a reduction in the rate of forced vital capacity (FVC) decline, the secondary analysis of SGRQ scores demonstrated improvements in the overall symptom burden, including cough, with modest enhancements in LCQ scores, indicating a positive effect on cough-related quality of life [61,62,63].

## 10. Novel Therapies for Intractable Cough

Two novel therapies targeting cough in ILD and IPF are P2X3 receptor antagonist/inhibitor and humidified high-flow therapy (HHFT). P2X3 receptor antagonists (Gefapixant, Filapixant, Silopixant) [64,65] were evaluated in a trial, where they reduced the cough frequency but with variable degrees of significant taste-related side effects, including dysgeusia and ageusia [66]. Gefapixant was approved in some countries but was not approved by the FDA by 2023. Long-term HHFT, though less studied, showed substantial improvement in cough severity in small case reports, with immediate effect upon commencement of treatment, by reducing the cough intensity through airway hydration and mucociliary clearance, with minimal side effects [67]. Both therapies offer promise but require further validation. 

## 11. IPF Treatments That Worsened/Did Not Improve Cough

### 11.1. Sodium Cromoglycate 

Inhaled sodium cromoglycate, once considered for treating chronic cough in various conditions, has shown mixed results in recent studies, particularly for IPF. Earlier research suggested the potential benefits of sodium cromoglycate in conditions like ACE-inhibitor cough and chronic lung diseases by inhibiting mast cell degranulation and reducing afferent C-fiber activity [68,69]. However, recent trials, such as the SCENIC trial, failed to demonstrate any significant efficacy of inhaled sodium cromoglycate (RVT-1601) in reducing the cough frequency in IPF patients [70,71]. This highlights its limited role in IPF-related cough management despite previous evidence in other respiratory conditions.

### 11.2. Phosphodiesterase 4B Inhibitor

In two recent studies, the phosphodiesterase 4B inhibitor BI 1015550 was evaluated for its impact on lung function and symptoms in patients with IPF. In a Phase 2 trial, BI 1015550 positively prevented the decline in forced vital capacity (FVC) over 12 weeks. Cough was monitored as a secondary outcome, but no significant changes in cough parameters were noted during this short-term study [72]. The Phase 3 FIBRONEER-IPF trial, which is currently active, will assess BI 1015550’s long-term impact, including changes in cough-related symptoms, as measured by the L-PF questionnaire [73].

### 11.3. Recombinant Human Pentraxin-2

In a long-term evaluation of recombinant human pentraxin-2 (rhPTX-2) for IPF, while the primary focus was on forced vital capacity (FVC) and 6 min walk distance (6MWD), cough was also monitored as a secondary outcome. Cough was one of the more frequently reported treatment-emergent adverse events (TEAEs), occurring in 27% of patients during the open-label extension period. The study noted no significant changes in cough-related outcomes as measured by the LCQ over time, with the LCQ total score showing little change from baseline to Week 128 [74].

### 11.4. Lysophosphatidic Acid 1 Antagonist

In a study on Admilparant, an LPA1 antagonist, cough was noted as a treatment-emergent adverse event (TEAE). In the IPF cohort, cough occurred in 10.8% of patients receiving 60 mg of Admilparant, while in the PPF cohort, it occurred in 11.9% of those receiving the same dose. These rates were slightly higher than those observed in the placebo group (5.4% for IPF and 9.8% for PPF). As the study moves into Phase 3 trials, further evaluation of cough as a TEAE is expected to continue alongside the primary endpoints related to lung function, particularly given the role of cough in affecting patients’ quality of life [74]. Table 2 outlines potential novel therapeutic approaches for the management of cough in patients with chronic cough, with ILD and IPF included.

### 11.5. Cough Suppression Techniques

Cough suppression techniques have garnered increasing attention as a non-pharmacological intervention for managing chronic cough, particularly in conditions like ILD. Although research on cough suppression in ILD is more limited than in other respiratory conditions, the evidence supports the efficacy of behavioral therapies in reducing cough frequency and the improving quality of life of these patients.

A pivotal study by Vertigan et al. (2006) explored speech pathology interventions, including cough suppression techniques, in patients with chronic refractory cough and demonstrated significant improvements in cough-related quality of life and reductions in cough frequency [75]. Although this study primarily focused on chronic cough that is not related to ILD, it provided a foundation for applying these techniques to ILD populations. Cough suppression techniques focus on managing the mechanisms of cough reflex hypersensitivity. Cough suppression physiotherapy, consisting of education, counselling, and cough suppression techniques, aims to retrain this reflex through controlled breathing, relaxation exercises, and vocal cord training. These interventions can help reduce the frequency and severity of cough by addressing the heightened sensitivity of the airways, as shown in ref. [76], including in patients with ILD who suffer from chronic refractory cough [77,78].

A pertinent example comes from a recent case report on an 83-year-old female with hypersensitivity pneumonitis with a chronic cough for 18 months, where non-pharmacological cough control therapy was initiated as part of the treatment plan. She participated in a 4-week biweekly session facilitated by a physiotherapist and a speech–language pathologist. The sessions included an introduction and overview of chronic cough and cough suppression techniques, followed by breathing exercises, vocal exercises, and laryngeal relaxation techniques. This was the first case report exploring non-pharmacological cough control therapy in an ILD patient. Improvement was noted in the HRQOL questionnaire, in addition to cough severity and intensity.

Although it may not benefit all patients, the risk of harm with therapy is minimal to none and could thus be a key adjunct to the comprehensive management in motivated patients.

## 12. Conclusions

Chronic cough in ILD remains a challenging symptom to evaluate and manage due to its multifactorial nature and significant impact on quality of life and disease outcomes. The current assessment relies on patient-reported tools such as the LCQ, CQLQ, and VAS, which, while valuable, require further validation for ILD-specific use. Therapeutic options, including neuromodulators, antifibrotic agents, and GERD management, provide variable benefits but often fall short of achieving comprehensive symptom control. Emerging treatments like P2X3 receptor antagonists and behavioral interventions, including cough suppression techniques, offer promising avenues for improvement. An individualized treatment focusing on a multidisciplinary approach that integrates robust evaluation tools and a combination of pharmacological and non-pharmacological strategies is essential for advancing the care of patients with ILD-related chronic refractory cough. This review not only highlights the potential updated treatment options that are available for managing cough in ILD/IPF but also underscores the lack of robust evidence supporting these approaches. There is a need for longer-term studies on opiates, which have shown strong short-term therapeutic potential, exploring the role of duloxetine in ILD/IPF cough, as well as larger RCTs exploring pharmacological and surgical treatments for GERD associated with ILD/IPF. Additionally, research specifically focusing on the role of physiotherapy in ILD/IPF patients is warranted. We eagerly anticipate the development and evaluation of novel agents targeting cough in ILD/IPF patients.

## Figures and Tables

**Table 1:** Studies evaluating the role and efficacy of opioid therapy in managing cough among patients with IPF.

Author	Article	Journal	Year	Result	Intervention
Zhe Wu, et al. [36]	PACIFY COUGH	*The Lancet Respiratory Medicine*	2024	In patients with cough related to IPF, low-dose controlled-release morphine significantly reduced objective cough counts over 14 days compared to placebo.	5 mg oral morphine twice daily.
Toby M. Maher, et al. [37]	Nalbuphine Tablets for Cough in Patients with IPF	*NEJM Evidence*	2023	NAL ER reduced cough in individuals with IPF.	Nalbuphine ER 27 mg once daily, up-titrated to 162 mg twice daily on day 16.

**Table 2:** Potential novel therapeutic approaches for the management of cough in patients with chronic cough, ILD and IPF.

Author	Article	Journal	Year	Result	Intervention
Aiko Niimi, et al. [64]	Randomised Trial of the P2X3 Receptor Antagonist Sivopixant for Refractory Chronic Cough	*ERS Publications*	2022	Sivopixant reduced objective cough frequency and improved HRQoL	Sivopixant 150 mg for 2 weeks
Christian Friedrich, et al. [65]	The P2X3 Receptor Antagonist Filapixant in Patients with Refractory Chronic Cough: An RCT	*BMC Respiratory Research*	2023	Filapixant significantly reduced cough frequency and severity and improved cough HRQoL	Filapixant doses ≥ 80 mg
Fernando J. Martinez, et al. [66]	Treatment of Persistent Cough in Subjects with IPD with Gefapixant, a P2X3 Antagonist, in a Randomized, Placebo-Controlled Clinical Trial	*Springer Nature*	2021	Gefapixant did not demonstrate a significant reduction versus placebo in cough by day 14	
Matthew Bricknell, et al. [67]	A Novel Therapy for Intractable Cough	*Respiralogy Case Reports*	2024	Case series of humidified high-flow therapy (HHFT) treatment of intractable chronic cough with IPF/ILD	
Fernando J. Martinez, et al. [70]	Phase 2B study of RVT-1601 for Chronic Cough in IPF (SCENIC Trial)	*American Journal of Respiratory and Critical Care Medicine*	2021	Treatment with inhaled RVT-1601 did not provide benefit over placebo for treatment of chronic cough in patients with IPF	Cromolyn Sodium 10, 40 and 80 mg
Luca Richeldi, et al. [71]	Trial of a Preferential PDE 4B Inhibitor for IPF (FIBRONEER-IPF)	*BMJ Open Respiratory Research*	2023	BI 1015550 (PDE4B inhibitor) prevented a decrease in lung function in patients with IPF	BI 1015550 9 mg BID, 18 mg BID

**Figure A1:**
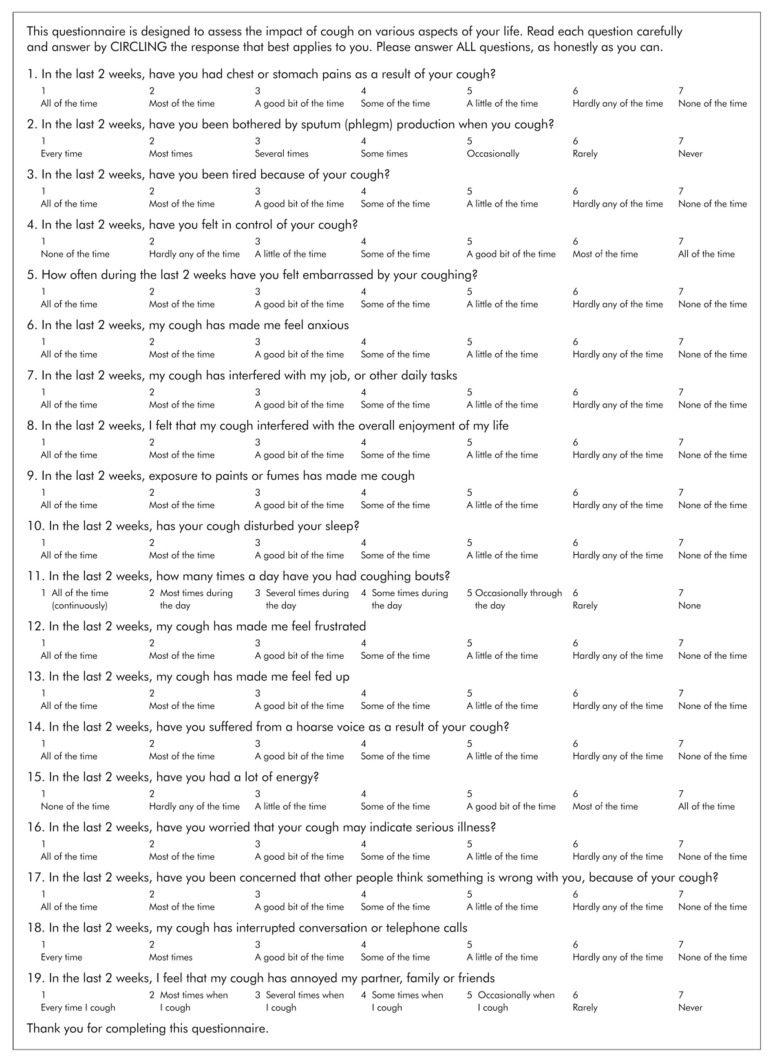
An example of the Leicester Cough Questionnaire. Available at LCQ. Published with permission.

**Figure A2:**
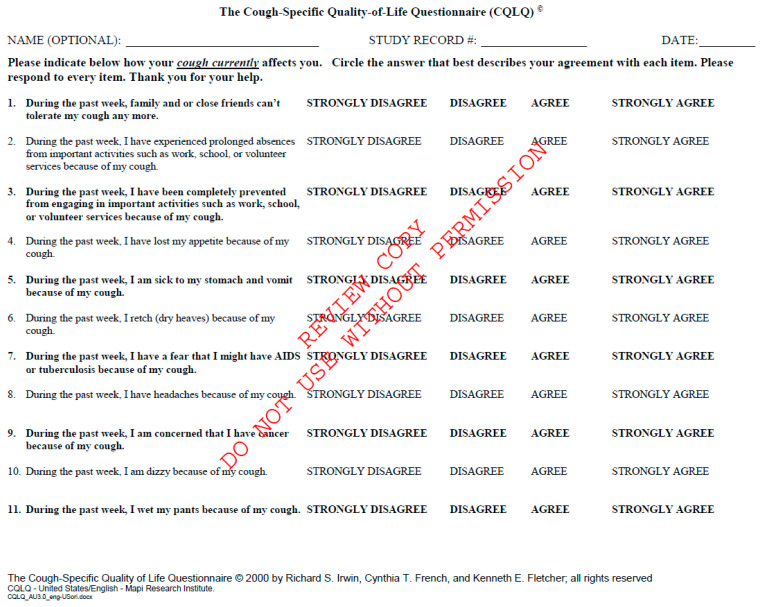

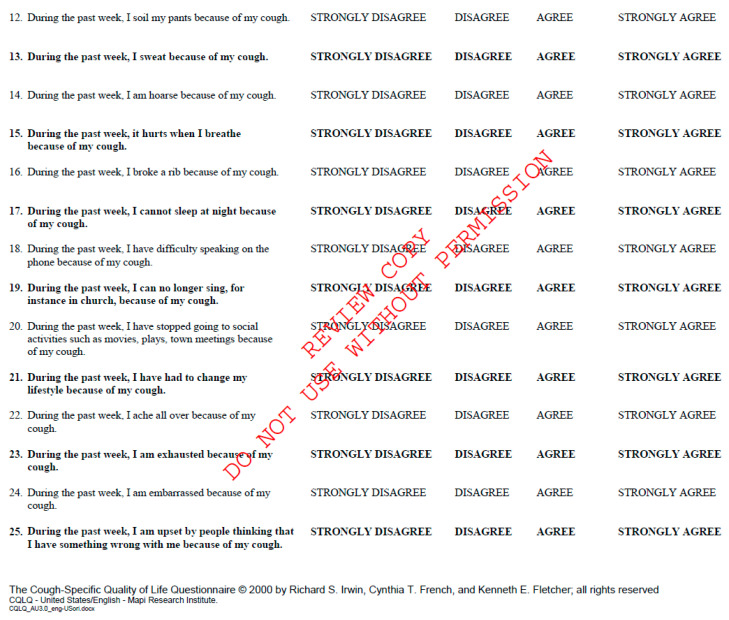

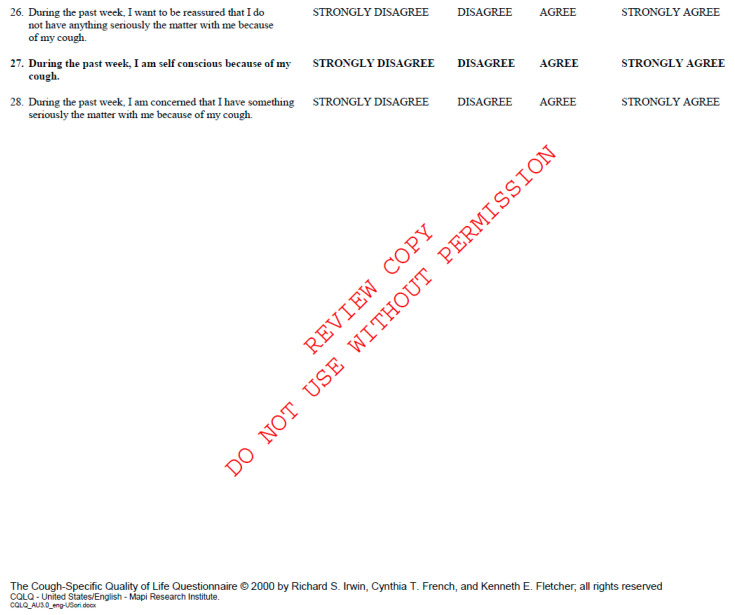
Cough Quality of Life Questionnaire. Available at CQLQ.

**Figure A3:**
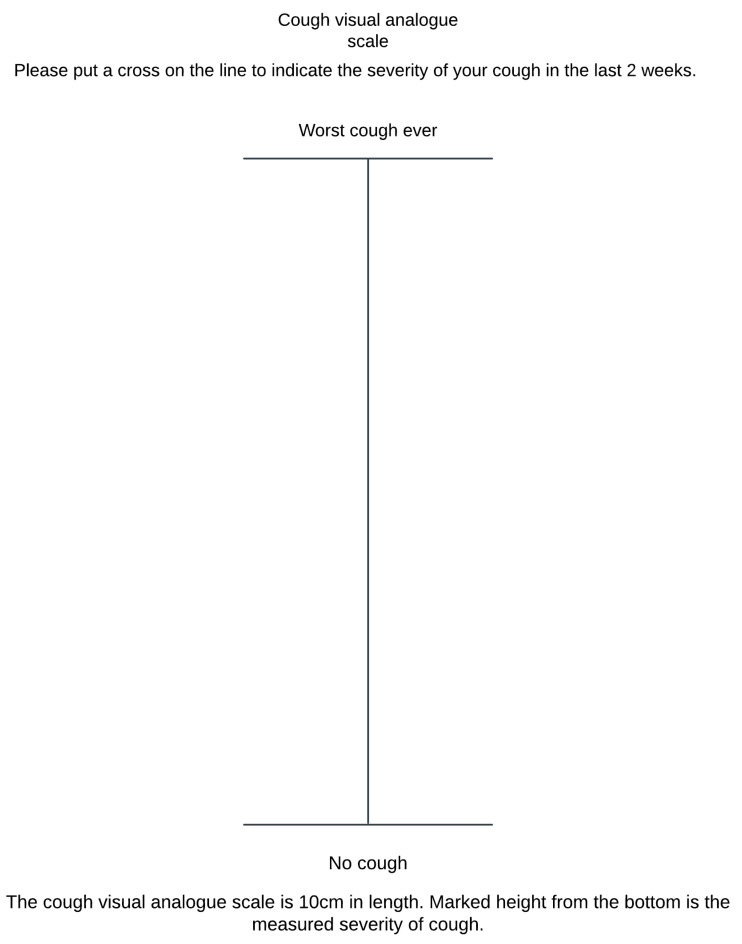
Cough Visual Analogue Scale. The vertical line should be 100 mm in length.

**Figure A4:**
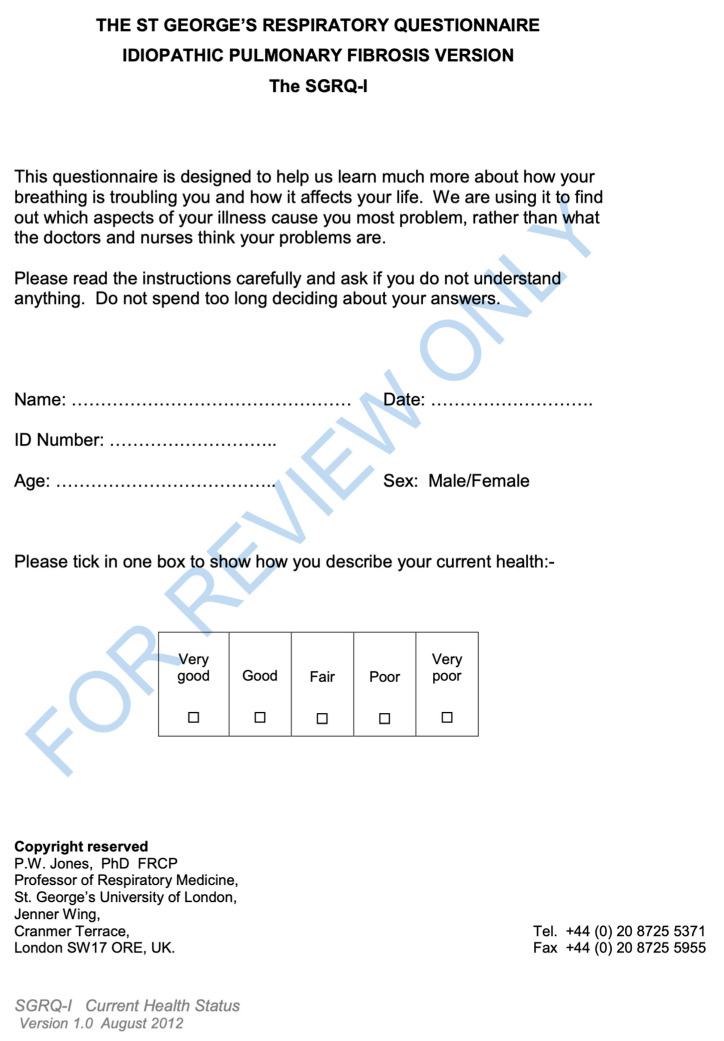

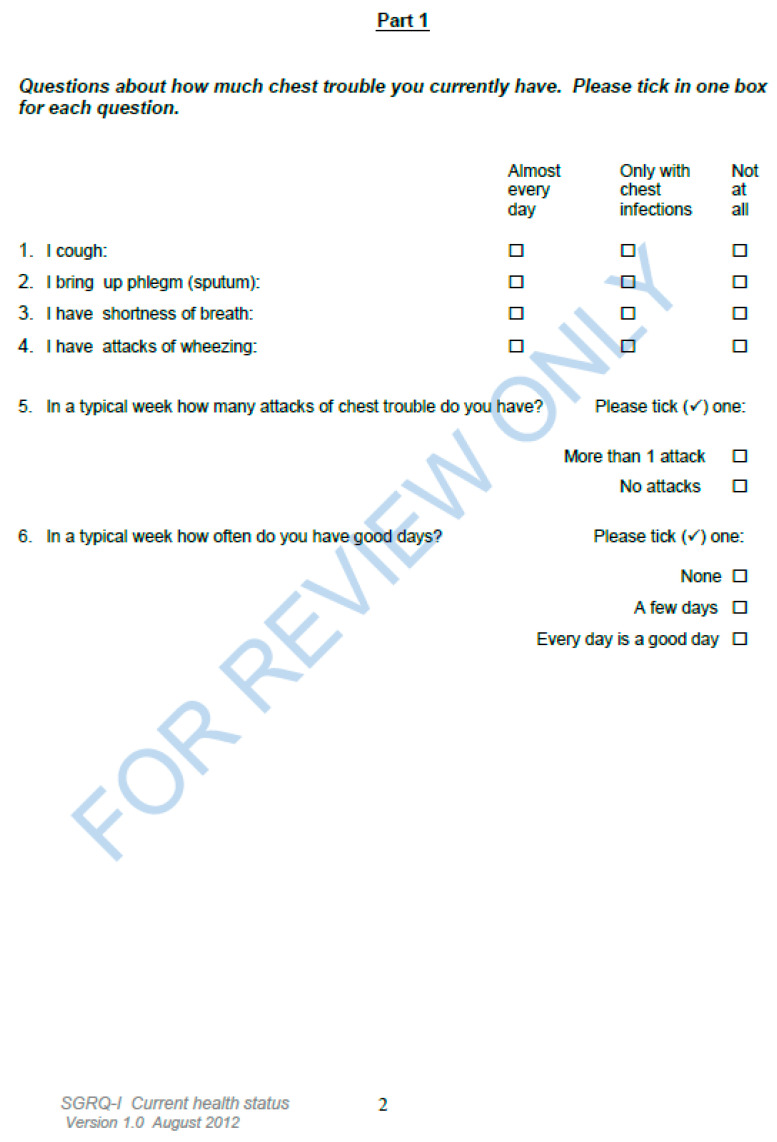

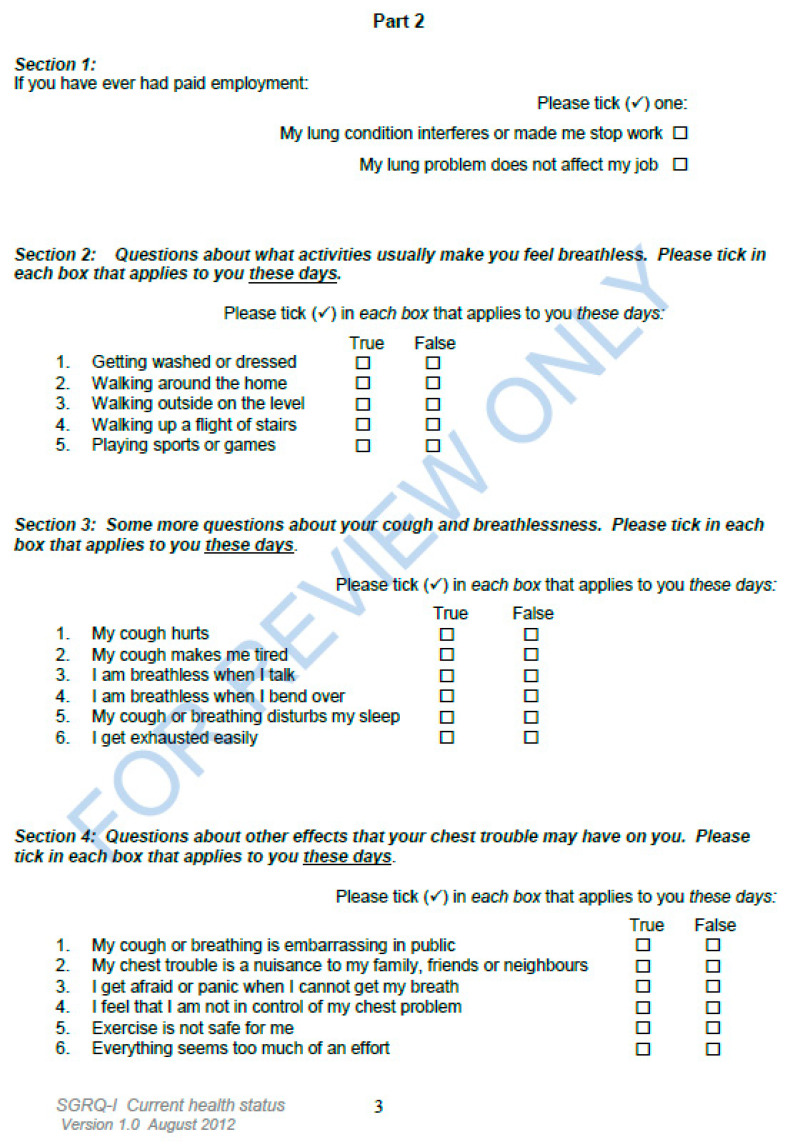

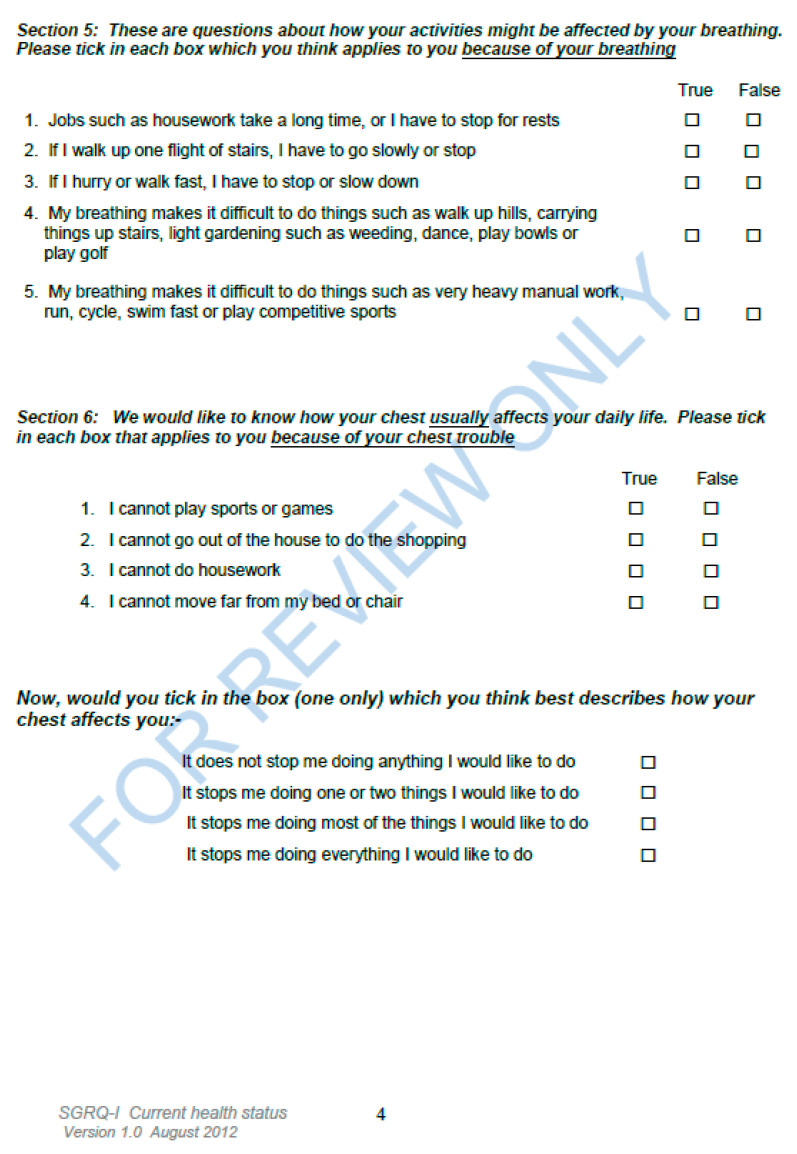
St. George’s Respiratory Questionnaire for IPF patients (document consists of four (4) pages). Published with permission from SGRQ.

**Figure A5:**
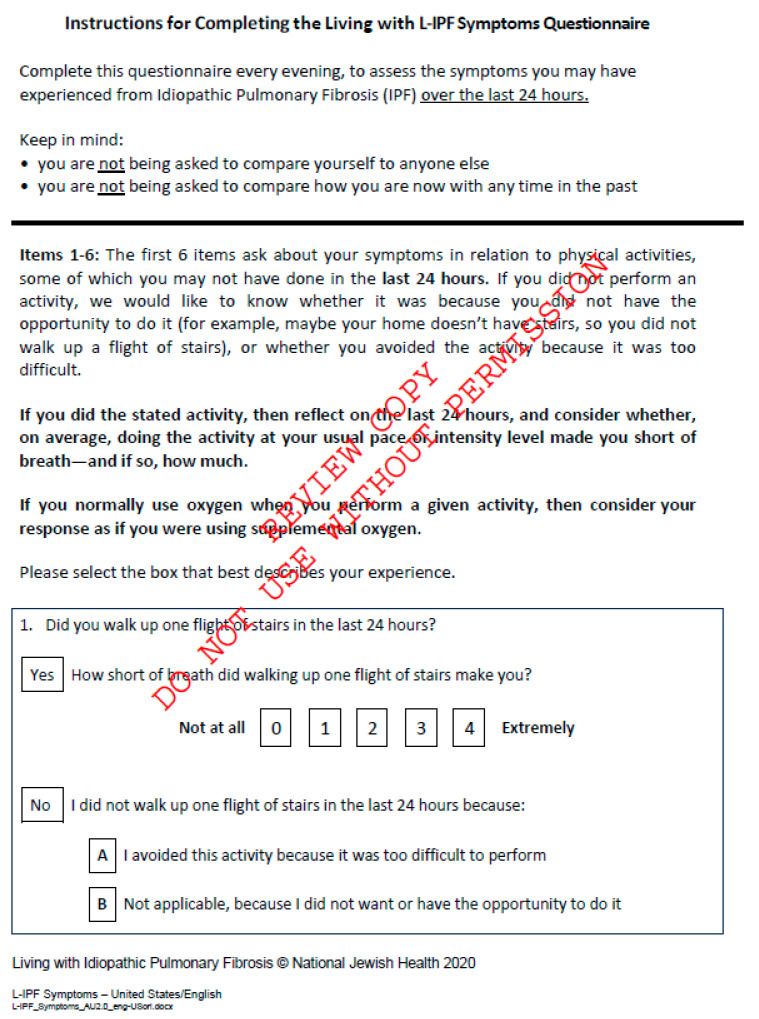

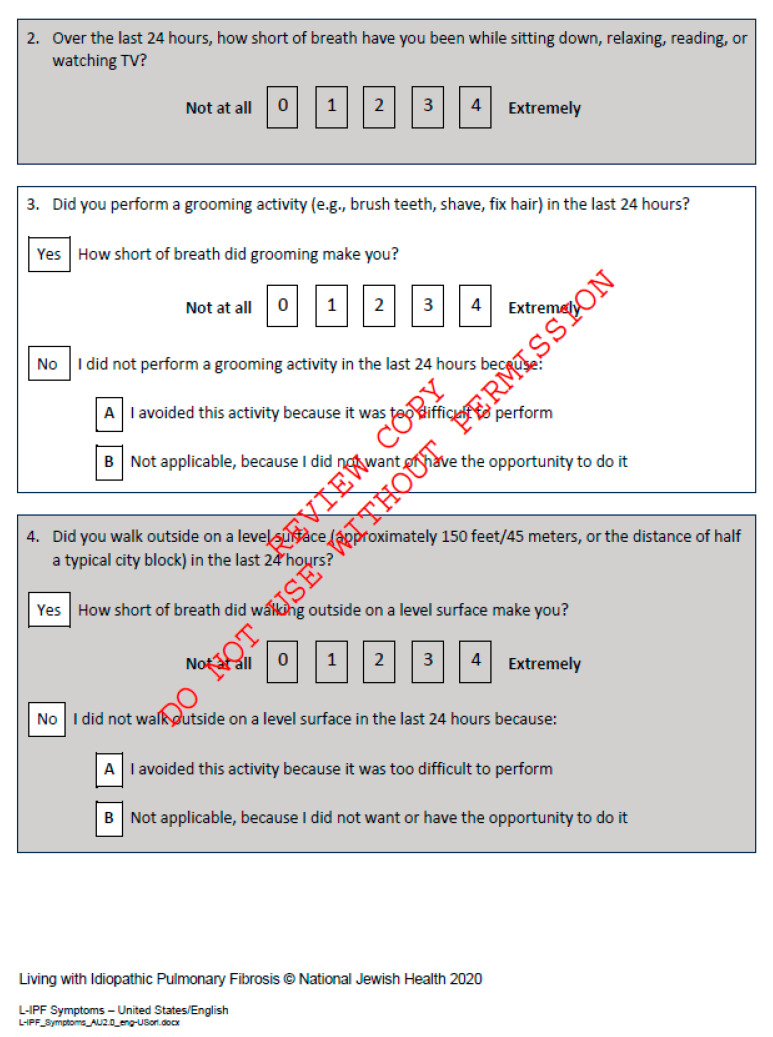

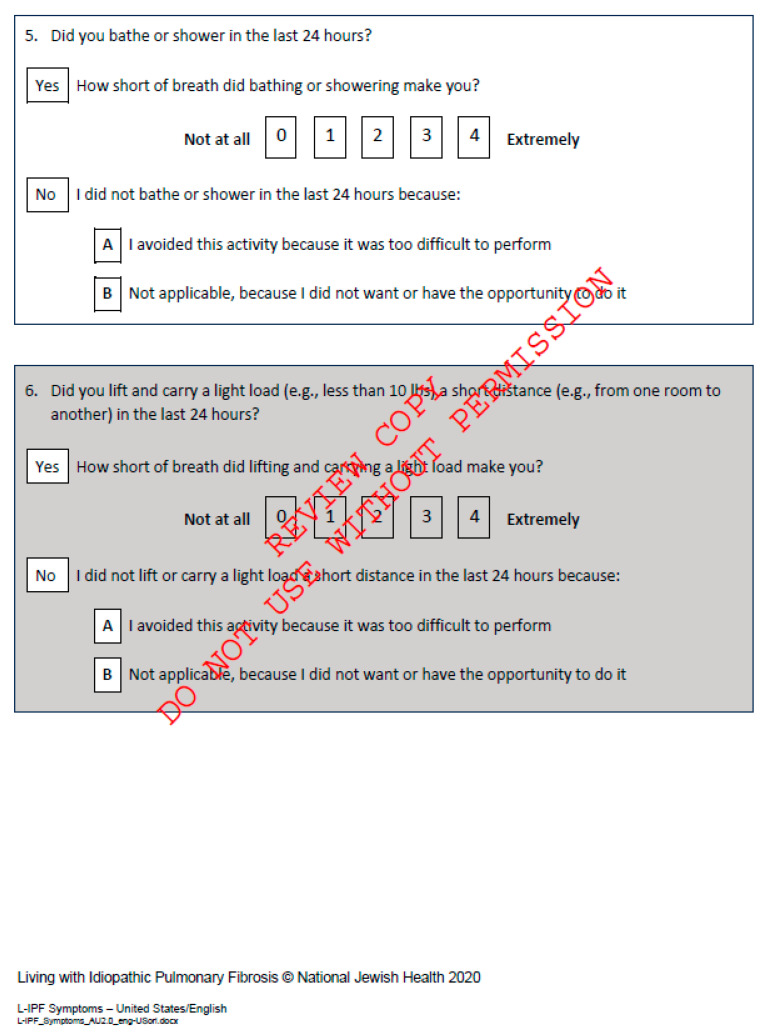

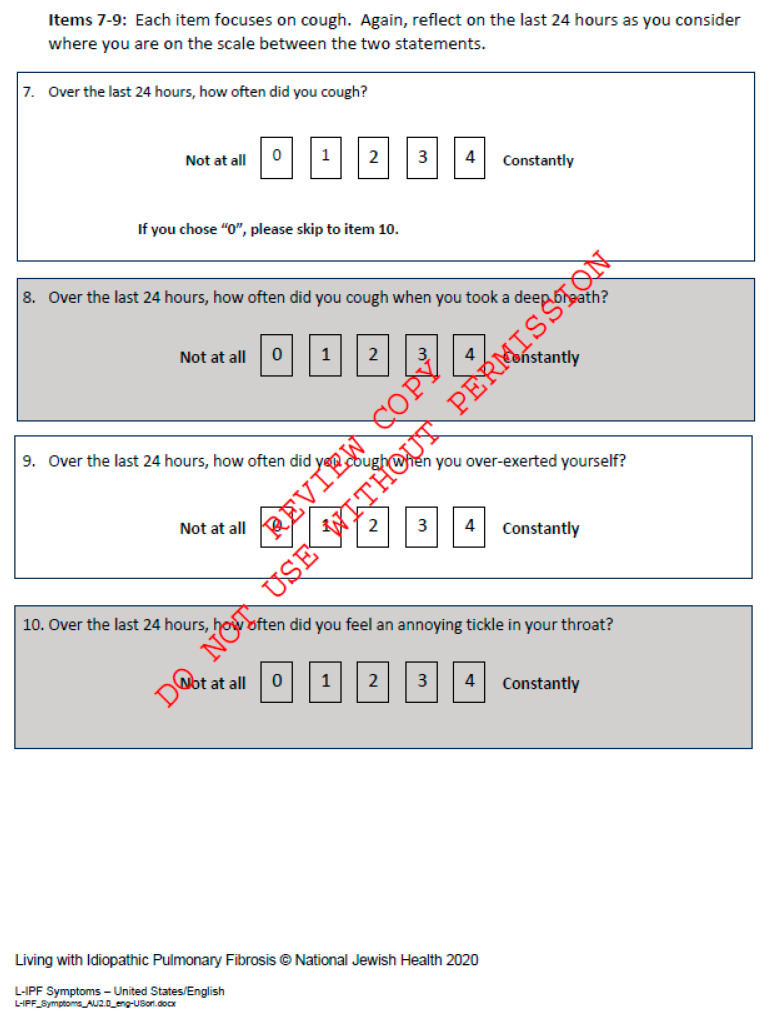

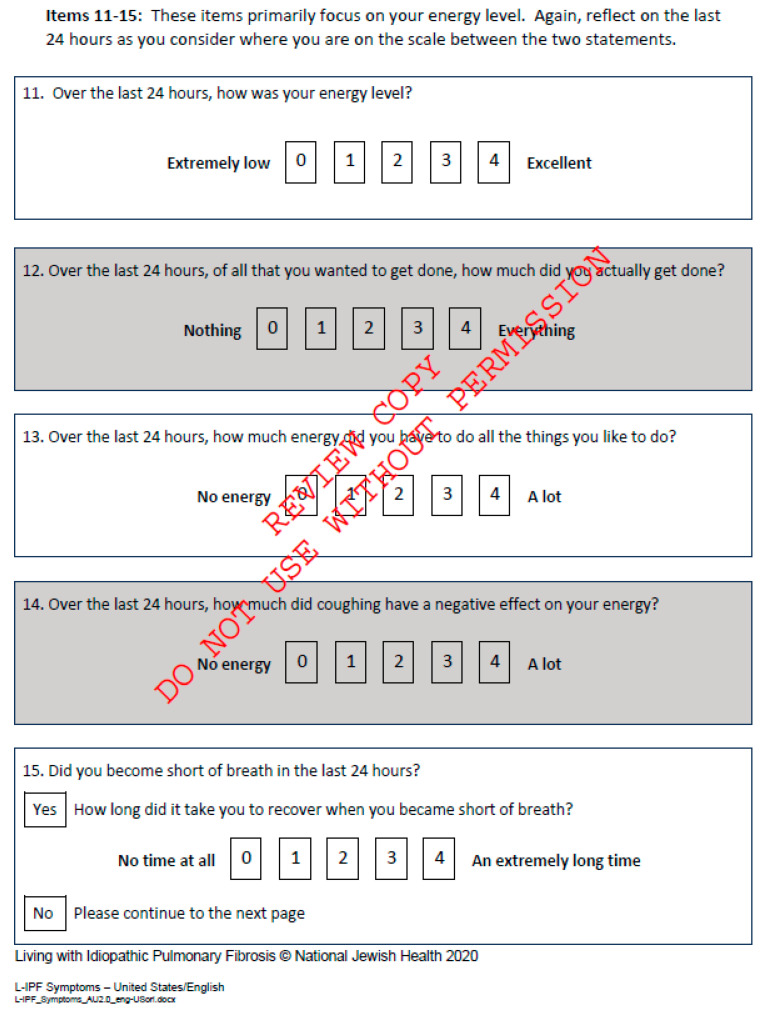

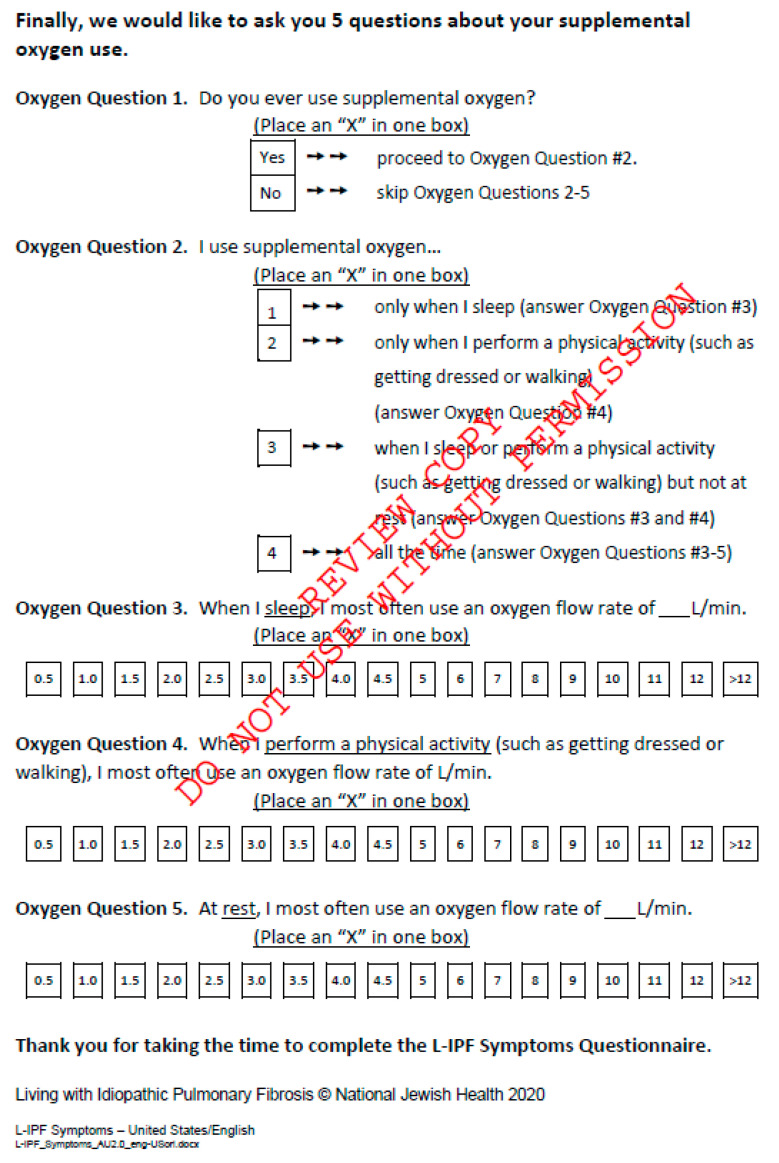
Living with Pulmonary Fibrosis Questionnaire, available at L-IPF.

## Data Availability

Not applicable.
